# *CERKL*, a Retinal Dystrophy Gene, Regulates Mitochondrial Transport and Dynamics in Hippocampal Neurons

**DOI:** 10.3390/ijms231911593

**Published:** 2022-09-30

**Authors:** Rocío García-Arroyo, Gemma Marfany, Serena Mirra

**Affiliations:** 1 Department of Genetics, Microbiology and Statistics, Universitat de Barcelona, Avda. Diagonal 643, 08028 Barcelona, Spain; 2 CIBERER, Instituto de Salud Carlos III, 28029 Madrid, Spain; 3 Institut de Biomedicina-Institut de Recerca Sant Joan de Déu (IBUB-IRSJD), Universitat de Barcelona, 08028 Barcelona, Spain; 4 DBGen Ocular Genomics, 08028 Barcelona, Spain

**Keywords:** *CERKL*, hippocampus, neurons, mitochondria, mitochondrial trafficking, mitochondrial dysfunction

## Abstract

Mutations in the *Ceramide Kinase-like* (*CERKL*) gene cause retinal dystrophies, characterized by progressive degeneration of retinal neurons, which eventually lead to vision loss. Among other functions, CERKL is involved in the regulation of autophagy, mitochondrial dynamics, and metabolism in the retina. However, *CERKL* is nearly ubiquitously expressed, and it has been recently described to play a protective role against brain injury. Here we show that *Cerkl* is expressed in the hippocampus, and we use mouse hippocampal neurons to explore the impact of either overexpression or depletion of CERKL on mitochondrial trafficking and dynamics along axons. We describe that a pool of CERKL localizes at mitochondria in hippocampal axons. Importantly, the depletion of CERKL in the *Cerkl*^*KD*/*KO*^ mouse model is associated with changes in the expression of fusion/fission molecular regulators, induces mitochondrial fragmentation, and impairs axonal mitochondrial trafficking. Our findings highlight the role of *CERKL*, a retinal dystrophy gene, in the regulation of mitochondrial health and homeostasis in central nervous system anatomic structures other than the retina.

## 1. Introduction

The central nervous system (CNS) is a high-metabolic-rate system, and its functionality is largely dependent on mitochondria. Mitochondria are the powerhouse of cells and actively participate in the regulation of cell respiratory mechanisms, metabolic processes, and energy homeostasis. Alterations in mitochondrial function can be due to genetic, physiological, or environmental cues and are frequently associated to the mitochondrial network remodeling and the alteration of mitochondrial dynamics, including fusion, fission, transport, interorganellar communication, and mitochondrial quality control [1,2]. Therefore, the failure of mitochondrial function and dynamics eventually leads to cell death and neurodegeneration [3].

The retina is the sensory system responsible for vision and belongs to the CNS, sharing with the brain a common developmental origin, cell-type composition, and anatomic and genetic features. Importantly, several well-defined neurodegenerative conditions originating from mitochondrial dysfunctions and affecting the brain and spinal cord have manifestations in the eye. Furthermore, various retinal pathologies caused by an impaired mitochondrial performance, share characteristics with other CNS pathologies [4].

*CERKL* is a retinal resilience gene whose mutations underlie retinitis pigmentosa and cone-rod dystrophy, two retinal dystrophies characterized by progressive vision loss due to photoreceptor degeneration [5]. The human *CERKL* gene is composed of 14 exons and presents an extremely high transcriptional complexity: it generates more than 20 transcripts in both human and mouse because of the use of multiple promoters and transcription start sites as well as alternative splicing events (Figure S1A) [6].

CERKL belongs to the CERK family, where the members share some protein domains, such as the pleckstrin homology domain, the diacylglycerol kinase domain, and the ATP binding site (Figure S1A) [7]. However, assays to show whether CERKL displays any kinase activity have been unsuccessful [8]. CERKL also displays two nuclear localization and two nuclear export signals (Figure S1B) [6]. These signals allow the protein to switch between the nucleus and the cytoplasm—where it localizes with endoplasmic reticulum and the Golgi apparatus [7].

In the human retina, we identified four main CERKL protein isoforms as a result of different alternative splicing events, although other protein isoforms might be also present (Figure S1B). The a isoform consists of 13 exons (532 amino acids); the b isoform contains an extra exon (4b), which is only found in humans (558 amino acids); the c isoform undergoes an alternative splicing event fusing exon 2 to exon 6 (419 amino acids); and the d isoform is the result of a splicing event fusing exon 3 to exon 6 (463 amino acids). Such a repertoire of protein isoforms displaying different domains suggests distinct cellular roles (Figure S1B) [7]. At the functional level, CERKL has been described as binding several neuronal calcium sensors [9] and sphingolipids [8], regulating general autophagy [10,11] and localizing at mitochondria in both immortalized and primary cell lines [11,12,13]. Moreover, *Cerkl* depletion is associated with alterations in mitochondrial size and distribution, and dysregulation of mitochondrial metabolism in the mammal retina [11].

CERKL is expressed in several tissues other than retina, such as neural tissues, kidney, lung, and testis. It has been recently proposed that CERKL can exercise a protective role against oxidative stress in a variety of contexts. Indeed, overexpression of CERKL protects retinal pigment epithelium from oxidative stress through the regulation of mitochondrial dynamics [13]. Moreover, CERKL enhances the survival of cutaneous squamous cell carcinoma challenged by oxidative stress [14]. CERKL overexpression has been also recently shown to alleviate the ischemia reperfusion induced nervous system injury by regulating SIRT1/PINK1/Parkin pathway [15]. Interestingly, the complete knockout of the *Cerkl* locus has been shown to be lethal at embryonic stages in homozygosis [16], highlighting the importance of *Cerkl* expression in vital organs or systems.

In this study, we explored the function of CERKL in regulating mitochondrial trafficking and dynamics in primary hippocampal neurons. We took advantages from the double heterozygote knockdown/knockout mouse model, *Cerkl^KD/KO^* (*KD*/*KO*), in which the *Cerkl* expression levels are highly reduced [16]. This mouse model showed a clear phenotype of progressive retinal neurodegeneration, but the effects of *Cerkl* depletion have not been yet studied in tissues other than the retina.

Our findings shed new insights into the molecular mechanisms regulating mitochondrial dynamics in neurons and may provide relevant information to develop prevention and treatment strategies to ameliorate not only retinal dystrophies but also neural pathologies, which frequently involve alteration of the oxidative stress response, and dysfunction of mitochondrial dynamics and bioenergetic homeostasis.

## 2. Results

### 2.1. CERKL Is Expressed in Hippocampal Neurons

It is well known that CERKL is highly expressed in the neuroretina where it plays an important role regulating stress response and mitochondrial dynamics and function [7,11,12]. Nevertheless, knowledge about CERKL function in other nervous tissues besides the retina has been growing lately [15]. Therefore, in this study we aimed to shed light on the function of CERKL related to mitochondrial dynamics in the brain.

**Figure 1:**
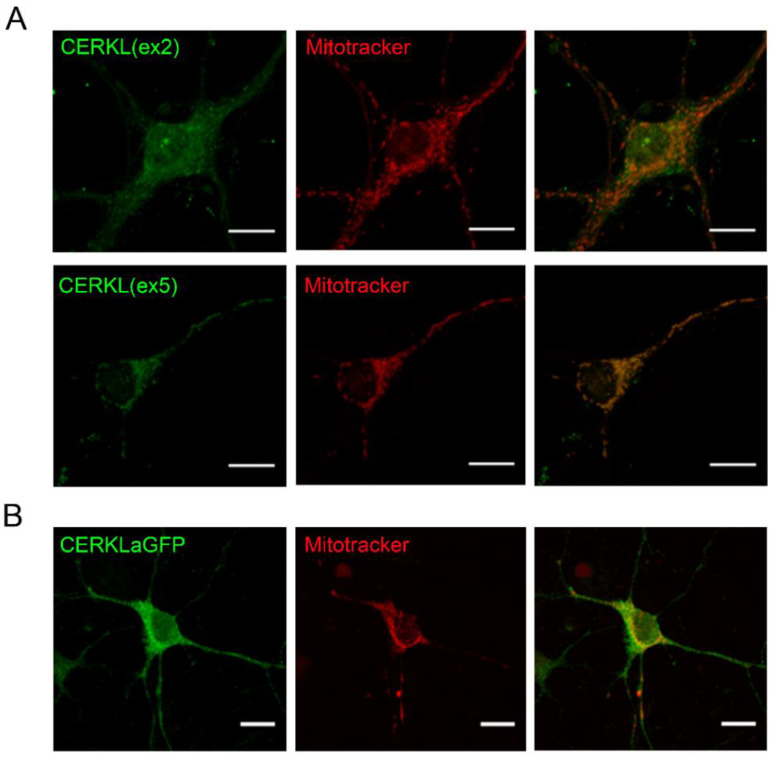
**CERKL is expressed in primary hippocampal neurons and partially localizes at mitochondria.** (**A**) CERKL is detected in primary hippocampal neurons (E16) by immunostaining with antibodies CERKL(ex2) or CERKL(ex5) (green). Mitotracker (red) is used to stain mitochondria. Note that CERKL isoforms containing exon 5 (detected with CERKL(ex5)) show higher localization at mitochondria. (**B**) Overexpression of CERKLa-GFP in primary hippocampal neurons shows partial localization of CERKLa (green) at mitochondria, which are stained with Mitotracker (red). Scale bars: 10 μm.

Hippocampi was selected as the source of brain neurons because it can be easily recognized and dissected from the mouse embryo brain, and the protocol for primary cell culture is well established. We first assessed whether *Cerkl* is expressed in hippocampal cells of the adult mouse brain through immunohistochemistry using an in-house antibody [16]. CERKL is detected in cornu ammonis 1–3 and the dentate gyrus (Figure S2). To further study the intracellular function of CERKL in hippocampal neurons, primary cell cultures from E16 embryos were analysed through immunocytochemistry using two different antibodies that recognize specific protein epitopes encoded in either exon 2 (ex2) or exon 5 (ex5) of the mouse *Cerkl* gene, as well as Mitotracker to stain mitochondria. We detected a differential CERKL subcellular localization with the two antibodies: while CERKL isoforms containing exon 2 were distributed diffusely within the neuron, including nucleus and cytoplasm, CERKL isoforms containing exon 5 showed higher localization at mitochondria (Figure 1A). In addition, we also transfected primary hippocampal neurons with hCERKLa-GFP (full length 532 aa isoform of human CERKL fused to GFP) and confirmed its expression and scattered localization in the nucleus, cytoplasm, and mitochondria (Figure 1B).

To sum up, CERKL is endogenously expressed in E16 and adult mouse hippocampi, suggesting it might play a role in both developing and in adult hippocampi. CERKL protein isoforms that contain the exon 5-encoded peptide show high colocalization with mitochondria.

### 2.2. Hippocampal Axons from Cerkl^KD/KO^ Mice Display Fragmented Mitochondria

**Figure 2:**
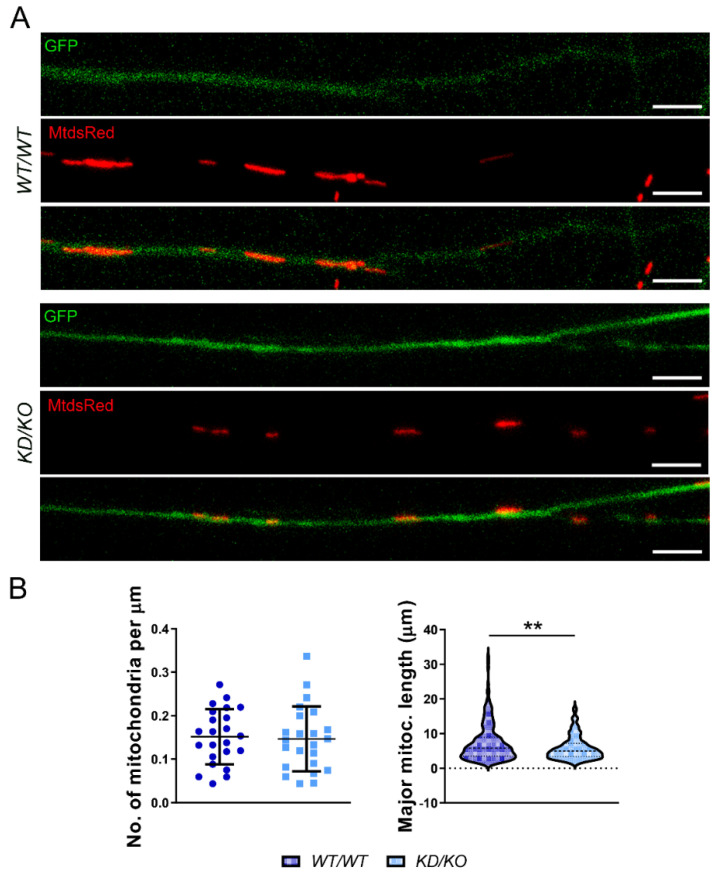
**CERKL depletion causes mitochondrial fragmentation in axons of hippocampal neurons**. (**A**) Axons from *WT*/*WT* and *KD*/*KO* primary hippocampal neurons were used to visualize and (**B**) quantify the mitochondrial density (mitochondria per μm) and mitochondrial length (μm). Hippocampal neurons were transfected with GFP to distinguish the axon (green) and MitoDsRed to stain mitochondria. Scale bars: 5 μm. Statistical analysis by *t*-test. *n* = 222–227 mitochondria from *n* = 23 axons per genotype. **: *p*-value ≤ 0.01.

Recent studies showed that the depletion of *CERKL* caused mitochondrial fragmentation in retinal ganglion cells and retinal pigment epithelium [11,13]. As *Cerkl* is expressed in hippocampi, we aimed to assess the effect of *Cerkl* depletion on mitochondrial morphology in this tissue, taking advantage of the double heterozygote knockdown/knockout *Cerkl^KD/KO^* mouse model (heretofore, *KD*/*KO*). Thus, *WT*/*WT* and *KD*/*KO* primary hippocampal neurons were cultured, and MitoDsRed-labelled individual mitochondria were analysed in their axons (Figure 2A). Analysis of mitochondrial morphological parameters revealed no changes in the number of mitochondria along the axon; instead, a significant decrease in major mitochondrial length of *KD*/*KO* neurons was detected (Figure 2B).

Overall, these results indicate that depletion of CERKL levels in *KD*/*KO* hippocampal axons causes mitochondrial fragmentation although it does not alter mitochondrial number (density).

### 2.3. Overexpression of CERKLa Does Not Affect Mitochondrial Morphology in Hippocampal Axons

**Figure 3:**
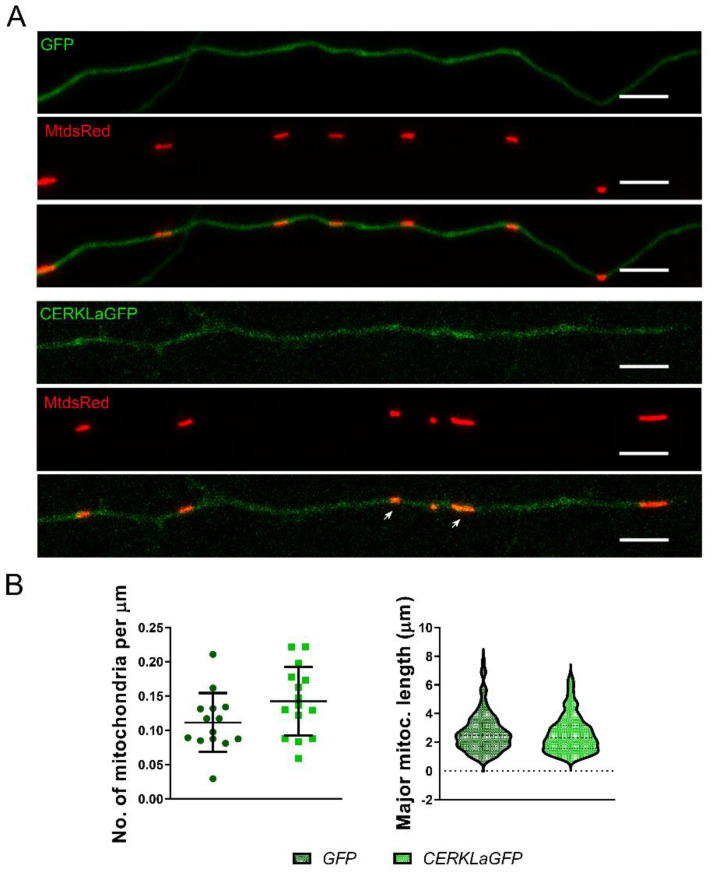
**Overexpression of CERKL does not alter mitochondrial morphology or size**. (**A**) GFP- (control) or CERKLa-GFP-transfected axons from *WT*/*WT* primary hippocampal neurons were used to quantify (**B**) the mitochondrial density and mitochondrial length. Mitochondria were detected by transfection with MitoDsRed. A pool of CERKL colocalizes with mitochondria (white arrows). Scale bar: 5 μm. Statistical analysis by *t*-test. *n* = 103–164 mitochondria from *n* = 14–15 axons per condition.

Once the effect of *Cerkl* depletion in mitochondrial morphology was evaluated using the *KD*/*KO* mouse model, we wondered whether overexpression of CERKL might also alter mitochondrial morphological parameters. To this end, *WT*/*WT* primary hippocampal neurons were transfected with either control GFP or CERKLa-GFP, as well as MitoDsRed to detect mitochondria. Although most CERKLa-GFP was distributed diffusely within the axon, a pool of the protein also localized at mitochondria (Figure 3A). Nonetheless, the analyses of mitochondrial morphology in the axons of the hippocampal cells overexpressing CERKL showed neither changes in mitochondrial density nor in major mitochondrial length compared to controls (Figure 3B).

In summary, overexpression of CERKLa-GFP did not alter mitochondrial density or size in hippocampal axons.

### 2.4. Mitochondrial Trafficking Is Reduced in KD/KO Hippocampal Axons

**Figure 4:**
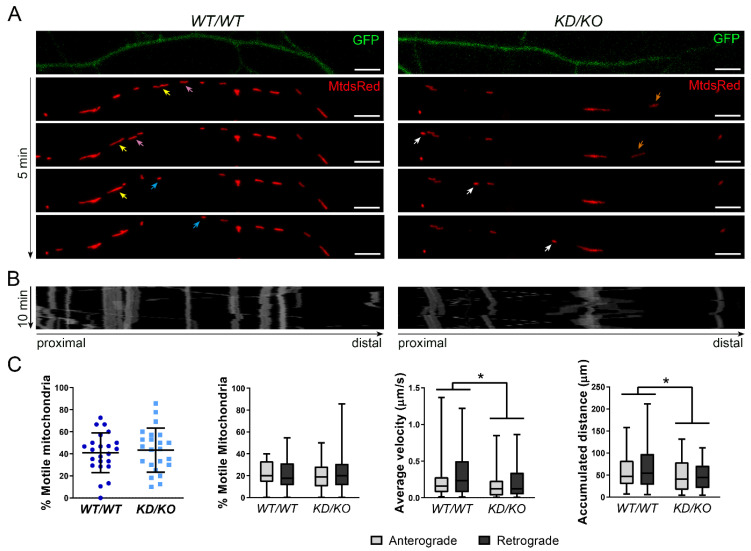
**Mitochondrial trafficking is reduced in *KD*/*KO* hippocampal neuron axons**. (**A**) Mitochondrial trafficking live imaging from *WT*/*WT* and *KD*/*KO* hippocampal cells (representative images of different timepoints over 5 min). Neurons were transfected with GFP to visualize the axon and MitoDsRed to label mitochondria. Yellow, pink, and orange arrows indicate mitochondria moving in anterograde direction. Blue and white arrows point mitochondria moving in anterograde direction. Scale bars: 5 μm. (**B**) Kymographs showing the path of each mitochondrion along the axons for 10 min. (**C**) CERKL depletion impairs mitochondrial trafficking in hippocampal axons. Percentage of total motile, anterograde, and retrograde mitochondria is not different between genotypes. However, average velocity and accumulated distance are reduced in *KD*/*KO* axons of hippocampal neurons. Statistical analysis by *t*-test and two-way ANOVA. *n* = 50–57 mitochondria from *n* = 23 axons per genotype. *: *p*-value ≤ 0.5.

Neuronal homeostasis is supported by proper mitochondrial transport from cell body to axons and dendrites, where ATP production and calcium buffering are required to guarantee correct neuronal function [17,18]. We aimed to assess whether mitochondrial trafficking was altered in *KD*/*KO* neurons. GFP and MitoDsRed were transfected to label individual axons and axonal mitochondria, respectively, in both *KD*/*KO* and *WT*/*WT* neurons. MitoDsRed transfection allows only a small number of neurons to be labelled, guaranteeing a precise analysis of anterograde and retrograde movements in single axons. Mitochondria from a segment of the axon were recorded over 10 min in live imaging experiments (Figure 4A) and mitochondrial trafficking was analysed through kymographs, which represent each mitochondrion movement through time (Figure 4B).

No differences between genotypes were found in the percentage of total moving mitochondria nor the percentage of mitochondria moving in anterograde or retrograde directions (Figure 4C). On the other hand, the analysis of the mitochondrial average velocity and accumulated distance per mitochondria showed significant changes between genotypes, with a clear decrease of both parameters in mitochondria from *KD*/*KO* axons, independently of the direction of the movement (Figure 4C).

Altogether, these results indicate that depletion of CERKL in *KD*/*KO* mice alters mitochondrial trafficking in hippocampal axons, resulting in a reduction of velocity and accumulated distance in mitochondria moving in both, anterograde and retrograde directions, while preserving the total number and percentage of moving mitochondria.

### 2.5. CERKLa-Transfected Hippocampal Axons Do Not Display Mitochondrial Trafficking Alterations

**Figure 5:**
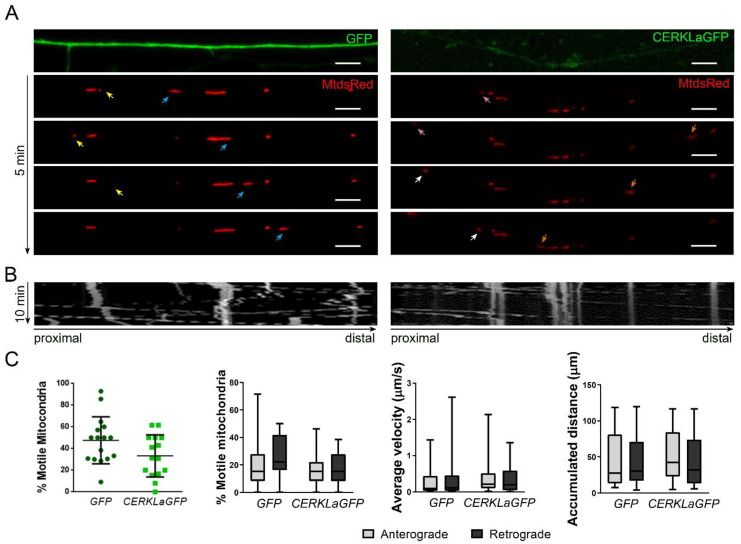
**Mitochondrial trafficking is not altered upon CERKL overexpression in hippocampal axons**. (**A**) Mitochondrial trafficking live imaging from GFP-(control) and CERKLa-GFP-transfected hippocampal axons (representative images of different timepoints over 5 min). Mitochondria were stained with MitoDsRed. Yellow, pink, and orange arrows indicate mitochondria moving in retrograde direction. Blue and white arrows point to mitochondria moving in anterograde direction. Scale bars: 5 μm. (**B**) Kymographs showing the path of each mitochondrion along the axons for 10 min. (**C**) CERKL overexpression does not alter mitochondrial trafficking in hippocampal axons. Percentage of total motile, anterograde, and retrograde mitochondria is not different between conditions. There are no differences in average velocity and accumulated distance in the axons of CERKLa-GFP-transfected primary hippocampal neurons. Statistical analysis by *t*-test and two-way ANOVA. *n* = 35–47 mitochondria from *n* = 14–15 axons per condition.

As CERKL depletion led to decreased mitochondrial trafficking in hippocampal axons, we also considered whether CERKL overexpression might also alter mitochondrial movement. To test this hypothesis, we recorded primary hippocampal neurons transfected with CERKLa-GFP and MitoDsRed (Figure 5A). Kymographs were again used to analyse mitochondrial trafficking from live imaging videos (Figure 5B). Notably, we did not observe any change in the percentage of total motile mitochondria in the axons of neurons transfected with CERKLa-GFP, and the balance between anterograde and retrograde mitochondria was maintained unaltered in comparison with control GFP-transfected axons. Moreover, neither average velocity nor accumulated distance in any direction were different in transfected versus control cells (Figure 5C).

Therefore, contrary to what happens with CERKL depletion, overexpression of CERKL does not alter the studied mitochondrial trafficking parameters in hippocampal axons.

### 2.6. Mitochondrial Fission Is Increased in KD/KO Hippocampi

**Figure 6:**
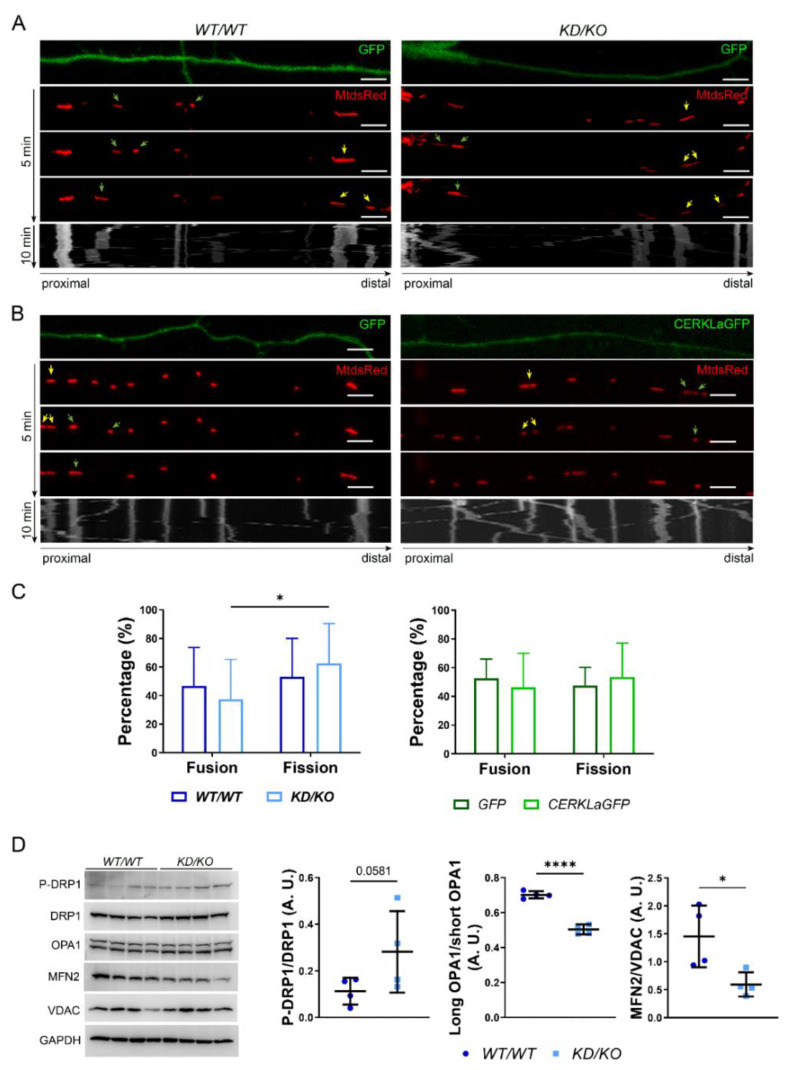
**Mitochondrial fission is increased in the axons of *KD*/*KO* hippocampal neurons**. Mitochondrial trafficking live imaging (representative images of different timepoints over 5 min) from axons of primary hippocampal neurons in order to analyse mitochondrial fusion (green arrows) and fission (yellow arrows) events in (**A**) CERKL overexpression conditions, comparing GFP-(control) and CERKLa-GFP-transfected neurons; and (**B**) CERKL depletion conditions, comparing *WT*/*WT* and *KD*/*KO* hippocampal axons. Mitochondria were stained with MitoDsRed. Scale bars: 5 μm. Kymographs showing the path of each mitochondrion along the axons for 10 min. (**C**) The percentage of fission events is increased in *KD*/*KO* hippocampal axons, whereas it remains unchanged in CERKLa-GFP-transfected hippocampal neurons, compared to their respective controls. Statistical analysis by two-way ANOVA. *n* = 23 axons per genotype, *n* = 14–15 axons per condition. (**D**) Mitochondrial fusion/fission proteins are altered in *KD*/*KO* hippocampi. Western blot analysis and quantification of P-DRP1, total DRP1, OPA1 and MITOFUSIN2 proteins in *WT*/*WT* and *KD*/*KO* hippocampi lysates show: an increase of P-DRP1/DRP1, and a decrease in long OPA1/short OPA1 and MFN2 levels in *KD*/*KO* samples. Statistical analysis by *t*-test. *n* = 4 animals per genotype. *: *p*-value ≤ 0.5; ****: *p*-value ≤ 0.0001.

In our experiments, we observed a reduction of mitochondrial size in *KD*/*KO* axons, suggesting alterations in mitochondrial fusion and/or fission. By taking advantage of recorded live imaging of *WT*/*WT* and *KD*/*KO* axons, we quantified fusion and fission events in each genotype (Figure 6A), as well as in GFP- or CERKL-aGFP-transfected neurons (Figure 6B). Notably, the percentage of fission events was significantly increased in *KD*/*KO* axons, while no changes were found upon CERKLa-GFP overexpression (Figure 6C), in concordance with the data obtained from mitochondrial morphology analyses (Figure 2 and Figure 3).

To obtain further insight into the molecular mechanisms regulating mitochondrial fission in *KD*/*KO* axons, we analysed the expression of mitochondrial fusion/fission key regulators in *WT*/*WT* and *KD*/*KO* hippocampal lysates. *KD*/*KO* hippocampi showed an increase in P-DRP1/DRP1 levels, although they did not reach statistical significance (Figure 6D). Notably, the ratio between the long and short OPA1 isoforms was significantly different due to an increase in the short OPA1 isoform in *KD*/*KO*, as MFN2 levels were also significantly decreased, overall indicating impaired mitochondrial fusion in *KD*/*KO* hippocampi (Figure 6D).

Overall, the depletion of CERKL induces fragmentation of mitochondria in *KD*/*KO* axons. This increase in mitochondrial fragmentation may be explained by increased levels of the activated (phosphorylated) form of the fission positive regulator DRP1, as well as by changes in both the ratio between OPA1 isoforms (regulating the inner mitochondrial membrane fusion) and the decreased expression of MFN2 (a positive regulator of the outer mitochondrial membrane).

### 2.7. Oxidative Phosphorylation Chain Complexes Are Altered in KD/KO Hippocampal Axons

**Figure 7:**
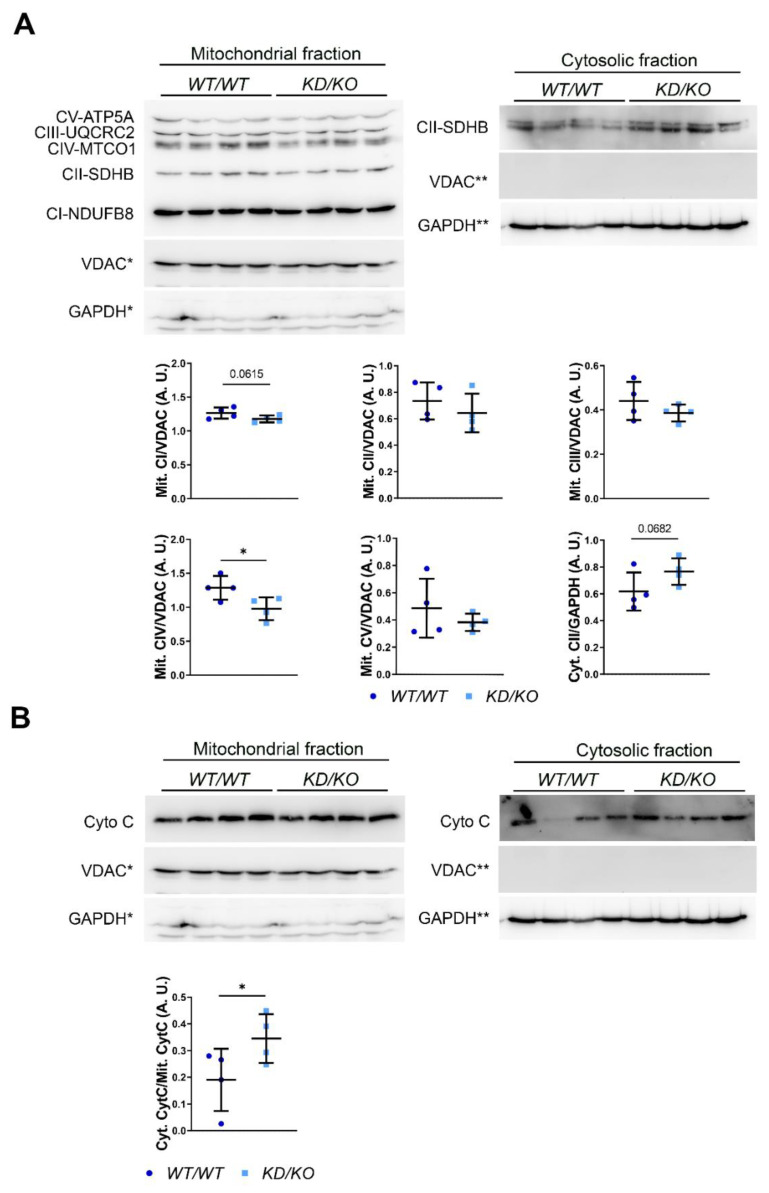
**OXPHOS protein content in mitochondrial fraction and cytochrome C release are differentially altered in *KD*/*KO* hippocampi**. (**A**) Western blot analysis and quantification of OXPHOS proteins in *WT*/*WT* and *KD*/*KO* cytosolic (Cyt) or mitochondrial fractions (Mit). VDAC and GAPDH are used as both loading and fraction enrichment controls. (**B**) Western blot analysis of cytochrome °C in mitochondrial and cytosolic fractions. Note that * or ** in both A and B, indicate that the same control has been used for different quantifications because immunodetections have been performed on the same membrane. Statistical analysis by *t*-test. *n* = 4 animals per genotype. *: *p*-value ≤ 0.5.

Our results show that upon CERKL depletion, hippocampal neurons displayed aberrant mitochondrial dynamics, including fission, fusion, and trafficking. Such alterations might also affect mitochondrial function [19]. Therefore, we evaluated the expression of the mitochondrial structural membrane protein VDAC and functional proteins of the OXPHOS system in WT/WT and KD/KO hippocampi by Western blot analysis. VDAC levels were significantly increased in KD/KO hippocampi, indicating an increase in mitochondrial mass. Additionally, quantification of OXPHOS complexes showed a significant decrease in the levels of CI-NDUFB8, CIII-UQCRC2 and CIV-MTCO1 per mitochondrial mass in KD/KO, whereas CII-SDHB and CV-ATP5A remained unaltered, suggesting differential regulation of functional and structural mitochondrial proteins (Figure S3). To shed further light on the changes in OXPHOS protein expression/localization at mitochondria, we performed mitochondria/cytosol fractionation experiments and checked the levels of both mitochondrial and cytosolic OXPHOS proteins. As expected, we found almost all the OXPHOS proteins (CI-NDUFB8, CIII-UQCRC2, CIV-MTCO1 and CV-ATP5A) exclusively in the mitochondrial fraction, where their expression is also differentially downregulated in *KD*/*KO* compared with *WT*/*WT* samples (Figure 7A). As in whole cell lysates, CV-ATP5A remained unaltered also in the mitochondrial fraction (Figure 7A). CII-SDHB, whose expression was found unaltered in whole cell lysates, was found in both mitochondrial fraction—where its expression tends to decrease in *KD*/*KO*—and in the cytosolic fraction—where its expression tends to increase in *KD*/*KO* (Figure 7A). Finally, we analyzed the cytochrome C release from mitochondria to cytosol compartment as a critical event related to mitochondrial integrity and apoptosis triggering. We found a statistically significant increase in the ratio between cytosolic and mitochondrial cytochrome C in *KD*/*KO* hippocampi (Figure 7B), reinforcing the notion of mitochondrial damage in the *KD*/*KO* tissue.

## 3. Discussion

To date, mutations in the *CERKL* gene have only been associated with retinal dystrophies, such as retinitis pigmentosa and cone-rod dystrophy [5,20], although recent studies have shown its potential role in other not-related-to-retina tissues and pathologies [14,15]. Although CERKL specific function still remains undetermined, there are several studies with evidence suggesting it plays a crucial role regulating homeostasis and survival of photoreceptors and retinal neurons acting as a resilience gene against apoptosis and regulating mitochondrial health in front of oxidative stress [7,12,13]. Correct mitochondrial function and dynamics are essential to maintain neuronal homeostasis and viability. In fact, in view of the high energy demand of the CNS, mitochondrial dysfunction is associated with the onset and progression of many neurodegenerative disorders [21]. Hence, in this work we aimed to assess CERKL implication on mitochondrial health in CNS tissues expressing *Cerkl* other than the retina, such as hippocampus.

Mitochondrial trafficking is an essential process that underlies the proper subcellular distribution of mitochondria from the soma, where they are produced, to axons and dendrites, to meet subcellular energetic demands and ensure the correct function and survival of neurons [17,22]. Here we describe, for the first time, that depletion of *Cerkl* impairs mitochondrial trafficking in neurons. More specifically, *KD*/*KO* hippocampal cell axons displayed an unchanged number of motile mitochondria, with a reduction in average velocity and accumulated distance of both anterogradely and retrogradely moving mitochondria (Figure 4C). These data suggest that CERKL is involved in regulating efficiency of mitochondrial trafficking and movement, rather than directional mitochondrial recruitment on microtubules. On the other hand, CERKLa overexpression does not affect these parameters of mitochondrial trafficking, pointing out that the relevant issue is to maintain sufficient functional CERKL protein upon a threshold rather than to maintain CERKL levels within a strict range. In fact, although the overexpression of several mitochondrial trafficking key regulators is known to potentiate mitochondrial trafficking in neurons [23,24,25], not always does protein overexpression result in a complementary physiological reverse effect from those obtained in down-regulation studies [26,27]. On the other hand, *CERKL* produce several protein isoforms [7], and we cannot discard the situation that the overexpression of distinct isoforms could differentially affect mitochondrial trafficking in neurons. Furthermore, different protein isoforms of CERKL may be expressed in brain due to tissue-specific mechanisms of alternative splicing regulation [28]. In line with this hypothesis, we show that different pools of protein isoforms containing exon 2 or exon 5 may localize at mitochondria in hippocampal neurons, with a strong localization of the isoforms containing the peptide encoded in exon 5 (including isoforms containing both exons). Given that the CERKL protein does not include any mitochondrial localization signal, we believe that the ATP-binding site encoded by exon 5 might promote association to mitochondria at basal level and/or in response to certain stimuli. Importantly, the most prevalent RP mutation (R283X) is located in exon 5, highlighting the importance of mitochondrial localization of CERKL. However, also isoforms including exon 2 presented a partial colocalization with the mitochondrial marker. This colocalization may derive from isoforms including both exons 2 and 5. Indeed, the PH domain encoded by exon 2 might also mediate recruitment of CERKL to mitochondria, since PH domains are involved in recruiting proteins to different membrane compartments. Considering the importance of correct mitochondrial transport for maintaining neuronal function and viability [29], our results provide new insight into the function of CERKL in maintaining neuronal homeostasis in the CNS.

Mitochondrial network is a highly dynamic structure in which mitochondria constantly undergo fusion and fission events to maintain mitochondrial health [19]. In mammalian cells, fission/fusion events are mainly mediated by several large dynamin-related GTPase proteins, including optic dominant atrophy 1 (OPA1), conserved dynamin-related GTPase (DRP1), and conserved dynamin-related GTPase mitofusion (MFN1 and MFN2) [3]. In this study we further characterized the effects of *Cerkl* down-regulation on mitochondrial morphology in hippocampal neurons. Our results showed a significant decrease in mitochondrial size in *KD*/*KO* hippocampal axons (Figure 2B), in concordance with previous studies that described mitochondrial fragmentation upon *Cerkl* depletion in different retinal cells, including neurons and retinal pigment epithelium [11,13]. In healthy cells, the frequency of mitochondrial fission and fusion events is equal in order to maintain mitochondrial number and morphology [19,30]. Nevertheless, *KD*/*KO* hippocampal neurons displayed a higher rate of fission events (Figure 6C). In agreement with that, we found an imbalance of the molecular machinery that regulates fusion and fission, namely down-regulation of MFN2, increase of DRP1 phosphorylation and reduction of long OPA1 isoform (Figure 6D), which prompts to decreased fusion and increased fission. Mitochondrial fusion–fission imbalance compromises mitochondrial health, and it is particularly relevant in environmental or genetic stress conditions [30]. Indeed, fusion rescues stress by allowing functional mitochondria to complement dysfunctional mitochondria by diffusion and sharing of components between organelles [31]. On the other hand, fission can be associated with the segregation of dysfunctional mitochondria that need to be eliminated throughout mitophagy [32]. Our data in hippocampal neurons are in accordance with our previous finding obtained in *KD*/*KO* retinal cells (photoreceptors, ganglion cells, and retinal pigment epithelium), where mitochondria are fragmented, with a consequent severe dysfunction in mitochondrial respiration and metabolism [11,13]. Then, our data further reinforce the important role of CERKL in mitochondrial network organization in neurons, although further research is recommended to identify the specific molecular mechanisms of the CERKL-mitochondria interaction.

**Figure 8:**
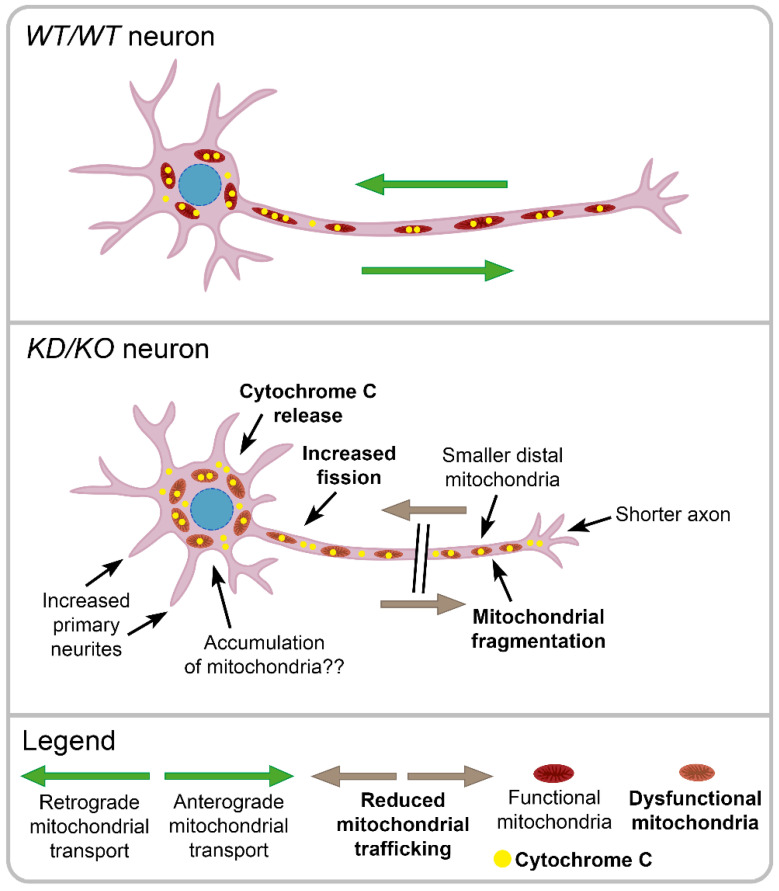
**Model recapitulating mitochondrial and morphological alterations in the *KD*/*KO* mouse retinal and hippocampal neurons**. *KD*/*KO* neurons show cytoarchitectural alterations, with an increased number of primary neurites and reduced axon length, compared to WT [16]. Besides, *KD*/*KO* neurons show dysfunctional mitochondrial (in orange) with an increase of mitochondrial fragmentation and fission events, a reduction of mitochondrial trafficking (brown arrows) and release of cytochrome C (in yellow) from mitochondria to cytosol. Our model proposes that *Cerkl* depletion causes altered distribution of mitochondria along the neuron, with an accumulation of mitochondria in the soma, an increase of fragmented mitochondria in the distal axon, and release of cytochrome C in the cytosol due to impaired mitochondria integrity (results from [11] and this work [highlighted in bold]).

In order to assure correct CNS function and facilitate synaptic transmission, the brain requires up to 25% of the body’s total glucose levels, which mainly undergoes mitochondrial oxidative phosphorylation (OXPHOS) [21,33]. We found a reduction in the levels of some OXPHOS chain complexes in *KD*/*KO* mitochondria (Figure 7A and Figure S3), which may involve mitochondrial dysfunction and neuronal bioenergetic impairment. Overall, our results indicate that *KD*/*KO* hippocampi display an altered OXPHOS chain structure/composition. Moreover, the protein levels of several OXPHOS subunits at the mitochondria are downregulated in *KD*/*KO* hippocampi. As for the CII-SDHB subunit, a fraction of which has been found to be retained in the cytosol, this change may be due to an altered transport of the protein at mitochondria. These results are in concordance with the deficiency in mitochondrial oxygen consumption in the retinas of *KD*/*KO* mice [11]. Notably, mitochondrial mass was increased in *KD*/*KO* hippocampi, although mitochondrial density in the axon fragments analysed did not appear altered (Figure 2B); probably the mitochondrial trafficking impairment results in decreased transport from the soma to the axons (Figure 4), in accordance with that reported in *KD*/*KO* retinal ganglion cells, where only smaller and fragmented mitochondria reached distal axonal segments [11]. This suggests an accumulation of dysfunctional mitochondria in the soma due to trafficking alterations and probably to defects in mitophagy [11], which also may explain the cytoarchitectural changes observed in retinal ganglion cells (increased number of neurites and shortened axons) [16]. Moreover, we observed an increased release of mitochondrial cytochrome C in cytosol, again supporting that the mitochondrial integrity is impaired in *KD*/*KO* hippocampi (Figure 7B). Therefore, all the phenotypic effects on mitochondrial dynamics, content, and metabolism due to *Cerkl* down-regulation are clearly conditioning neuron homeostasis and function. Our proposed model based on these results is summarized in Figure 8.

*CERKL* has been proposed as a resilience gene against oxidative stress and its protective function is usually triggered by a challenge. In absence of stress conditions, overexpression of CERKL does not alter the mitochondrial network, in contrast, it clearly protects mitochondria from oxidative stress [13]. In this work, cultured neurons were not challenged by oxidative stress conditions, and thus it is not surprising that CERKL overexpression did not alter any of the studied mitochondrial parameters (Figure 3, Figure 5, and Figure 6C). In addition, CERKL overexpression might be contributing to other pathways besides mitochondrial dynamics, such as the formation of RNA stress granules [34], regulation of autophagy [10], and apoptosis prevention [7]. However, CERKL depletion affects the mitochondrial network and makes cells more vulnerable to stress conditions [11,13]. We detected endogenous expression of CERKL in both embryonic and adult hippocampi from *WT*/*WT* mice (Figure 1 and Figure S1). Embryonic development is a process that involves multiple changes where mitochondrial proper function is crucial in determining cell fate and maintaining cell growth and survival [33,35]. In this context, the observed effects of *Cerkl* depletion in embryonic hippocampi suggest that CERKL may be playing such an important role in CNS development, that it would provide a rationale for embryonic lethality upon homozygous total deletion of *Cerkl* in mouse [16].

Interestingly, although CERKL plays a protective role against oxidative stress in different tissues such as epidermis and brain [14,15], *CERKL* mutations have been reported to solely affect the retina so far. Each tissue is especially vulnerable to different stress events and relies on distinct resilience molecular mechanisms [36]. In this context, CERKL seems to be a key stress regulator for retinal health whereas it might not be that relevant in other regions of the CNS. Indeed, the contribution of CERKL to different tissue-specific pathways may explain the absence of brain phenotype. Therefore, further studies in tissues other than the retina might shed light on other phenotypic alterations due to *CERKL* mutations.

## 4. Materials and Methods

### 4.1. Animal Experimentation

WT and *Cerkl^KD/KO^* mice (C57BL/6J) were bred and housed in the animal research facilities at the University of Barcelona. Animals were provided with food and water ad libitum and maintained in a temperature-controlled environment in a 12/12 h light–dark cycle. Animal experiments were performed according to the ARVO statement for the use of animals in ophthalmic and vision research, as well as the regulations of the Ethical Committee for Animal Experimentation (AEC) of the Generalitat de Catalunya (protocol C-449/18), according to the European Directive 2010/63/EU and other relevant guidelines.

### 4.2. Genomic DNA and Genotyping by PCR

DNA for genotyping was extracted from ear punches. Primers for genotyping and PCR conditions are described in [16].

### 4.3. Cell Culture and Transfections

Hippocampi were carefully dissected from WT and *Cerkl^KD/KO^* mouse brains (E16 embryos) in PBS containing 3% glucose. Then, the samples were treated with trypsin (Invitrogen, Carlsbad, CA, USA) and DNAse (Roche Diagnostics, Indianapolis, IN, USA) and physically dissociated into single neurons. Neurons were plated on glass coverslips or 35-mm *Fluorodish* plates (World Precision Instruments Inc), coated with 0.5 mg/mL poly-L-lysine (Sigma-Aldrich, St. Louis, MO, USA) and incubated in neurobasal medium (Gibco, Grand Island, NY, USA) containing 2 mM glutamax, 120/mL penicillin, 200/mL streptomycin, and B27 supplement (Invitrogen, Waltham, MA, USA). Cells were maintained at 37 °C in the presence of 5% CO_2_ and were cultured for between 5 and 6 days. Hippocampal cultures at 4 DIV were transfected with Lipofectamine 2000 (Life Technologies, Carlsbad, CA, USA) according to the manufacturer’s guidelines.

In immunocytochemistry experiments on fixed cells, 1 μM MitoTracker™ Orange CMTMRos (Thermo Fisher Scientific, Rockford, IL, USA) was added to the neurons to stain mitochondria and incubated for 20 min at 37 °C before fixation.

### 4.4. Plasmid Vectors

CERKLa-GFP was obtained by cloning the coding region of hCERKL532 cDNA (NM_201548.4) in pEGFPN2 (BD Bioscience, NJ, USA), by using XhoI and BamHI restriction sites. Mitochondrial-targeted DsRed (mitoDsRed) was kindly provided by Prof. Eduardo Soriano (University of Barcelona, Barcelona, Spain).

### 4.5. Immunofluorescence

For immunocytochemistry, primary neurons were fixed in pre-chilled methanol at −20 °C for 10 min, washed in PBS (3 × 5 min), permeabilized in 0.2% Triton X-100 (St. Louis, MO, USA) in 1× PBS (20 min at RT), and blocked for 1 h in 10% Normal Goat Serum (Roche Diagnostics) in 1× PBS. Primary antibodies were incubated overnight at 4 °C in blocking solution. The primary antibodies used were CERKL(ex2) and CERKL(ex5), and were obtained in-house against epitopes encoded in exon 2 or exon 5 of the mouse *Cerkl* gene respectively [16]. After incubation, coverslips with cells were rinsed in 1× PBS (3 × 5 min), incubated with the corresponding secondary antibodies conjugated to Alexa Fluor 488 (Life Technologies, Grand Island, NY, USA) (1:500) at RT (1 h) in blocking solution. Nuclei were stained with DAPI (Roche Diagnostics, Indianapolis, IN, USA) (1:1000), washed again in 1× PBS (3 × 5 min), and mounted in Mowiol 4–88 (Merck, Darmstadt, Germany).

### 4.6. Tissue Processing and Histology

Adult *WT*/*WT* mice were anesthetized and perfused with 4% PFA in 0.1 M phosphate buffer (PB). Brains were carefully extracted, post-fixed overnight with 4% PFA in PB, cryoprotected with 30% sucrose in PBS, and frozen at −42 °C in isopentane. Frozen brain samples were sectioned in 30-μm coronal sections using a freezing microtome (Leica, Wetzlar, Germany). Free-floating sections were collected in cryoprotectant solution (85% glycerol, 100% ethylene glycol, 0.1 M PBS) and kept at −20 °C until use. For immunohistochemistry, frozen brain sections were permeabilized and incubated for 2 h at RT with 0.2% Triton-X-100, 1% BSA and 0.2 M glycine in PBS. The same solution was used for CERKL(ex2) primary antibody incubation, performed overnight at 4 °C, and Alexa Fluor 488 secondary antibody incubation, (Life Technologies, Grand Island, NY, USA) (1:500), performed for 2 h at RT. Nuclei were stained using DAPI (Roche Diagnostics, Indianapolis, IN, USA) (1:1000), and sections were mounted in Mowiol 4–88 (Merck, Darmstadt, Germany).

### 4.7. Western Blot

Hippocampi from adult WT and *Cerkl^KD/KO^* mice were carefully dissected and lysed in RIPA buffer [50 mM Tris, pH 7.4, 150 mM NaCl, 1 mM EDTA, 1% NP-40, 0.25% Na-deoxycholate, protease inhibitors (Complete Mini Protease Inhibitor Cocktail Tablets; Roche, Indianapolis, IN, USA)]. In mitochondria/cytosol fractioning experiments, we used the Cytochrome c Release Assay Kit (GeneTex, Alton Pkwy, Irvine, CA, USA, GTX85531), following the manufacturer’s instructions to obtain mitochondrial and cytosolic fraction. Proteins were separated by SDS-PAGE and transferred onto nitrocellulose membranes, which were blocked with 5% non-fat dry milk in tris-HCl-buffered saline (TBS) containing 0.1% Tween 20 and incubated overnight at 4 °C with primary antibodies. After incubation with horseradish peroxidase-labelled secondary antibodies for 1 h at RT, membranes were revealed with the ECL system (Lumi-Light Western Blotting Substrate, Roche, Indianapolis, IN, USA). Images were acquired by ImageQuant^TM^ LAS 4000 mini Image Analyser (Fujifilm, Tokyo, Japan) and quantified using ImageJ software. TUBULIN or GAPDH loading controls were used when needed. The primary antibodies used were: VDAC (Sigma-Aldrich, St. Louis, MO, USA, (Ab-5) (185–197); 1:7000), TUBULIN (Sigma-Aldrich, St. Louis, MO, USA, T5168, 1:1000), GAPDH (Abcam, Cambridge, UK, ab8245, 1:1000), Rodent Total OXPHOS Cocktail (MitoSciences, Eugene, OR, USA, 6 µg/mL), MITOFUSIN2 (Abcam, Cambridge, UK, ab56889, 1:1000), OPA1 (Proteintech, Rosemont, IL, USA, 27733-1-AP, 1:1000), DRP1 (Cell Signaling Technology, Danvers, MA, USA, 14647S, 1:1000), P-DRP1 (Cell Signaling Technology, Danvers, MA, USA, S616:3455S, 1:000), Anti-Cytochrome C antibody (GeneTex, Alton Pkwy, Irvine, CA, USA, GTX108585, 1:1000).

The secondary antibodies used were: HRP-labelled anti-mouse (Vector Labs, Mowry Ave Newark, CA, USA; P447-01, 1:2000) and anti-rabbit (Vector Labs, Mowry Ave Newark, CA, USA; P217-02, 1:2000).

### 4.8. Live Cell Imaging and Microscope Image Acquisition

In vivo imaging experiments were performed at 37 °C in an atmosphere of 5% CO_2_ with an LSM780 confocal microscope (Zeiss, Oberkochen, Germany) equipped with 40x (NA 1.3) and 63x (NA 1.4) oil objectives. All electronics were controlled through the ZEN software (Zeiss, Oberkochen, Germany). MitoDsRed-labelled mitochondria in axons were live imaged 1–2 days after transfection (5–6 DIV cultures). In mitochondrial tracking experiments, an axonal segment located approximately 90 to 160 µm distal to the soma was selected for live imaging. Z stacks of 7 images from the axonal region were taken every 6 s over 10 min using the mitoDsRed channel with 800 × 100 pixel resolution and an extra 2× digital zoom. Movies were processed using ImageJ software (http://imagej.nih.gov/ij/, accessed on 4 December 2017), and kymographs were generated by tracing axons in their z-projections. In kymographs, straight vertical lines were considered as static mitochondria, and motile mitochondria (non-straight vertical lines) were traced to evaluate their motility and directionality. The percentage of time in motion was calculated as the percentage of time a given mitochondrion (static or motile) spent moving at speed over 0.0083 µm/s towards the anterograde or retrograde direction and represented as an average. The percentage of motile mitochondria represents the relation between the number of motile and static mitochondria for each condition.

### 4.9. Mitochondrial Number and Length

Live neuronal cultures expressing mitoDsRed were imaged with a LSM780 (Zeiss, Oberkochen, Germany) confocal microscope equipped with a 63x oil objectives. Confocal images of the red (MtDsRed) channel were acquired, then number and length of mitochondria within the axon were quantified using an ImageJ software macro as described in [37] and standardized to the length of the axonal section imaged. Mitochondrial length represents the major axis length of mitochondria. Mitochondrial number and length were determined from axonal proximal segments of 14–23 neurons per condition.

### 4.10. Analysis of Mitochondrial Fusion and Fission Events

Events of fusion and fission were manually calculated from the same axons recorded in live cell imaging experiments. Both movies and kymographs were used for the quantification. The percentage of fusion or fission represents the relation between the number of fusion or fission events and the total number of fusion and fission events in each single recorded axon.

### 4.11. Statistical Analyses

Statistical analyses were performed using the two-tailed unpaired Student’s *t*-test and two-way ANOVA. When data did not follow a normal distribution, non-parametric Mann–Whitney test was used to determine the statistical significance. ROUT test was used to determine statistical outliers (Q = 1%). Calculations were performed with GraphPad Prism statistical software, version 6 (GraphPad6 Software Inc., San Diego, CA, USA). N is shown at each figure legend. Statistical significance was set with a *p*-value ≤ 0.05, (*: *p*-value ≤ 0.05, **: *p*-value ≤ 0.01, ***: *p*-value ≤ 0.005, ****: *p*-value ≤ 0.001). Data are expressed as standard deviation (SD).

## 5. Conclusions

Overall, in this work we determined that CERKL is not only involved in mitochondrial morphology and function in neurons, but also in mitochondrial trafficking regulation, contributing to the intricate network that regulates mitochondrial health in neurodegenerative diseases. Therefore, *CERKL* might play an important role as a resilience gene regulating neuronal homeostasis and viability in the brain during embryonic development and adult stages. We propose *CERKL* as a potential candidate gene contributing to neurological pathologies due to its implication in mitochondrial dynamics and resilience to stress in the CNS.

## Data Availability

The data presented in this study are available in this article and supplementary material.

## Supplementary Materials

The following supporting information can be downloaded at: https://www.mdpi.com/article/10.3390/ijms231911593/s1.

## References

[1] Muthuraman A., Ramesh M., Shaikh S.A., Aswinprakash S., Jagadeesh D. (2021). Physiological and Pathophysiological Role of Cysteine Metabolism in Human Metabolic Syndrome. Drug Metab. Lett..

[2] Mirra S., Marfany G. (2019). Mitochondrial Gymnastics in Retinal Cells: A Resilience Mechanism Against Oxidative Stress and Neurodegeneration. Advances in Experimental Medicine and Biology.

[3] Yang D., Ying J., Wang X., Zhao T., Yoon S., Fang Y., Zheng Q., Liu X., Yu W., Hua F. (2021). Mitochondrial Dynamics: A Key Role in Neurodegeneration and a Potential Target for Neurodegenerative Disease. Front. Neurosci..

[4] London A., Benhar I., Schwartz M. (2013). The retina as a window to the brain—From eye research to CNS disorders. Nat. Rev. Neurol..

[5] Aleman T.S., Soumittra N., Cideciyan A.V., Sumaroka A.M., Ramprasad V.L., Herrera W., Windsor E.A.M., Schwartz S.B., Russell R.C., Roman A.J. (2009). CERKL mutations cause an autosomal recessive cone-rod dystrophy with inner retinopathy. Investig. Ophthalmol. Vis. Sci..

[6] Garanto A., Riera M., Pomares E., Permanyer J., de Castro-Miro M., Sava F., Abril J.F., Marfany G., Gonzalez-Duarte R. (2011). High transcriptional complexity of the retinitis pigmentosa CERKL gene in human and mouse. Investig. Ophthalmol. Vis. Sci..

[7] Tuson M., Garanto A., Gonzalez-Duarte R., Marfany G. (2009). Overexpression of CERKL, a gene responsible for retinitis pigmentosa in humans, protects cells from apoptosis induced by oxidative stress. Mol.Vis..

[8] Garanto A., Mandal N.A., Egido-Gabás M., Marfany G., Fabriàs G., Anderson R.E., Casas J., Gonzàlez-Duarte R. (2013). Specific sphingolipid content decrease in Cerkl knockdown mouse retinas. Exp. Eye Res..

[9] Nevet M.J., Vekslin S., Dizhoor A.M., Olshevskaya E.V., Tidhar R., Futerman A.H., Ben-Yosef T. (2012). Ceramide kinase-like (CERKL) interacts with neuronal calcium sensor proteins in the retina in a cation-dependent manner. Investig. Ophthalmol. Vis. Sci..

[10] Hu X., Lu Z., Yu S., Reilly J., Liu F., Jia D., Qin Y., Han S., Liu X., Qu Z. (2019). CERKL regulates autophagy via the NAD-dependent deacetylase SIRT1. Autophagy.

[11] Mirra S., García-Arroyo R., Domènech E.B., Gavaldà-Navarro A., Herrera-Úbeda C., Oliva C., Garcia-Fernàndez J., Artuch R., Villarroya F., Marfany G. (2021). CERKL, a retinal dystrophy gene, regulates mitochondrial function and dynamics in the mammalian retina. Neurobiol. Dis..

[12] Li C., Wang L., Zhang J., Huang M., Wong F., Liu X., Liu F., Cui X., Yang G., Chen J. (2014). CERKL interacts with mitochondrial TRX2 and protects retinal cells from oxidative stress-induced apoptosis. Biochim. Biophys. Acta-Mol. Basis Dis..

[13] García-Arroyo R., Gavaldà A., Villarroya F., Marfany G., Mirra S. (2021). Overexpression of CERKL Protects Retinal Pigment Epithelium Mitochondria from Oxidative Stress Effects. Antioxidants.

[14] Meyer J.M., Lee E., Celli A., Park K., Cho R., Lambert W., Pitchford M., Gordon M., Tsai K., Cleaver J. (2021). CERKL is upregulated in cutaneous squamous cell carcinoma and maintains cellular sphingolipids and resistance to oxidative stress. Br. J. Dermatol..

[15] Huang S., Hong Z., Zhang L., Guo J., Li Y., Li K. (2022). CERKL alleviates ischemia reperfusion-induced nervous system injury through modulating the SIRT1/PINK1/Parkin pathway and mitophagy induction. Biol. Chem..

[16] Domènech E.B., Andres R., López-Iniesta M.J., Mirra S., García-Arroyo R., Milla S., Sava F., Andilla J., Alvarez P.L., De La Villa P. (2020). A New Cerkl Mouse Model Generated by CRISPR-Cas9 Shows Progressive Retinal Degeneration and Altered Morphological and Electrophysiological Phenotype. Investig. Ophthalmol. Vis. Sci..

[17] Sheng Z.H., Cai Q. (2012). Mitochondrial transport in neurons: Impact on synaptic homeostasis and neurodegeneration. Nat. Rev. Neurosci..

[18] Chamberlain K.A., Sheng Z.H. (2019). Mechanisms for the maintenance and regulation of axonal energy supply. J. Neurosci. Res..

[19] Detmer S.A., Chan D.C. (2007). Functions and dysfunctions of mitochondrial dynamics. Nat. Rev. Mol. Cell Biol..

[20] Tuson M., Marfany G., Gonzàlez-Duarte R. (2004). Mutation of CERKL, a Novel Human Ceramide Kinase Gene, Causes Autosomal Recessive Retinitis Pigmentosa (RP26). Am. J. Hum. Genet..

[21] Trigo D., Avelar C., Fernandes M., Sá J., da Cruz e Silva O. (2022). Mitochondria, energy, and metabolism in neuronal health and disease. FEBS Lett..

[22] Schwarz T.L. (2013). Mitochondrial trafficking in neurons. Cold Spring Harb. Perspect. Med..

[23] MacAskill A.F., Kittler J.T. (2010). Control of mitochondrial transport and localization in neurons. Trends Cell Biol..

[24] Fang D., Yan S., Yu Q., Chen D., Yan S.S. (2016). Mfn2 is required for mitochondrial development and synapse formation in human induced pluripotent stem cells/hiPSC derived cortical neurons. Sci. Rep..

[25] Norkett R., Modi S., Birsa N., Atkin T.A., Ivankovic D., Pathania M., Trossbach S.V., Korth C., Hirst W.D., Kittler J.T. (2016). DISC1-dependent regulation of mitochondrial dynamics controls the morphogenesis of complex neuronal dendrites. J. Biol. Chem..

[26] Wang W., Li L., Lin W.L., Dickson D.W., Petrucelli L., Zhang T., Wang X. (2013). The ALS disease-associated mutant TDP-43 impairs mitochondrial dynamics and function in motor neurons. Hum. Mol. Genet..

[27] Serrat R., Mirra S., Figueiro-Silva J., Navas-Pérez E., Quevedo M., López-Doménech G., Podlesniy P., Ulloa F., Garcia-Fernàndez J., Trullas R. (2014). The Armc10/SVH gene: Genome context, regulation of mitochondrial dynamics and protection against Aβ-induced mitochondrial fragmentation. Cell Death Dis..

[28] Aísa-Marín I., García-Arroyo R., Mirra S., Marfany G. (2021). The alter retina: Alternative splicing of retinal genes in health and disease. Int. J. Mol. Sci..

[29] Vanhauwaert R., Bharat V., Wang X. (2019). Surveillance and transportation of mitochondria in neurons. Curr. Opin. Neurobiol..

[30] Karbowski M., Youle R.J. (2003). Dynamics of mitochondrial morphology in healthy cells and during apoptosis. Cell Death Differ..

[31] Liu Y.J., McIntyre R.L., Janssens G.E., Houtkooper R.H. (2020). Mitochondrial fission and fusion: A dynamic role in aging and potential target for age-related disease. Mech. Ageing Dev..

[32] Sun N., Youle R.J., Finkel T. (2016). The Mitochondrial Basis of Aging. Mol. Cell.

[33] Pérez M.J., Quintanilla R.A. (2017). Development or disease: Duality of the mitochondrial permeability transition pore. Dev. Biol..

[34] Fathinajafabadi A., Pérez-Jiménez E., Riera M., Knecht E., Gonzàlez-Duarte R. (2014). CERKL, a retinal disease gene, encodes an mRNA-binding protein that localizes in compact and untranslated mRNPs associated with microtubules. PLoS ONE.

[35] Khacho M., Harris R., Slack R.S. (2019). Mitochondria as central regulators of neural stem cell fate and cognitive function. Nat. Rev. Neurosci..

[36] Kim J.M., Kim H.G., Son C.G. (2018). Tissue-Specific Profiling of Oxidative Stress-Associated Transcriptome in a Healthy Mouse Model. Int. J. Mol. Sci..

[37] Cherubini M., Puigdellívol M., Alberch J., Ginés S. (2015). Cdk5-mediated mitochondrial fission: A key player in dopaminergic toxicity in Huntington’s disease. Biochim. Biophys. Acta-Mol. Basis Dis..

