# Supplementary material for: CERKL, a Retinal Dystrophy Gene, Regulates Mitochondrial Transport and Dynamics in Hippocampal Neurons
Source: Int J Mol Sci. 2022 Sep 30;23(19):11593. doi: 10.3390/ijms231911593 (PMC9570143; doi:10.3390/ijms231911593)
Supplement: Supplementary file 1 [file ijms-23-11593-s001.zip › ijms-1922056-supplementary.pdf]

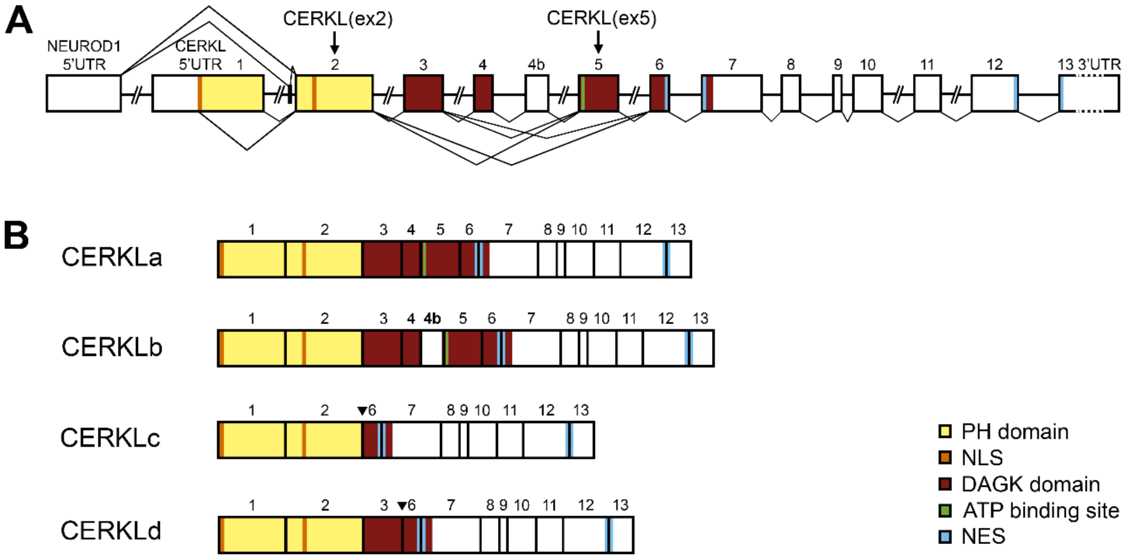

Figure S1. Schematic representation of the genomic structure and transcription of *CERKL* and some encoded protein retinal isoforms. (**A**) The human *CERKL* gene consists of 14 exons, uses multiple transcriptional start sites and undergoes multiple alternative splicing events. The position of the encoded domains in exons 2 and 5 used for antibody production are also indicated. (**B**) In the human retina, *CERKL* generates at least 4 protein isoforms as a result of alternative splicing events. Each of these isoforms displays different domains (PH, pleckstrin; DAGK, diacylglycerol kinase domain; NLS, nuclear localization signals; NES, nuclear export signals). Black triangles in isoforms c and d indicate the position of alternatively spliced exon junctions.

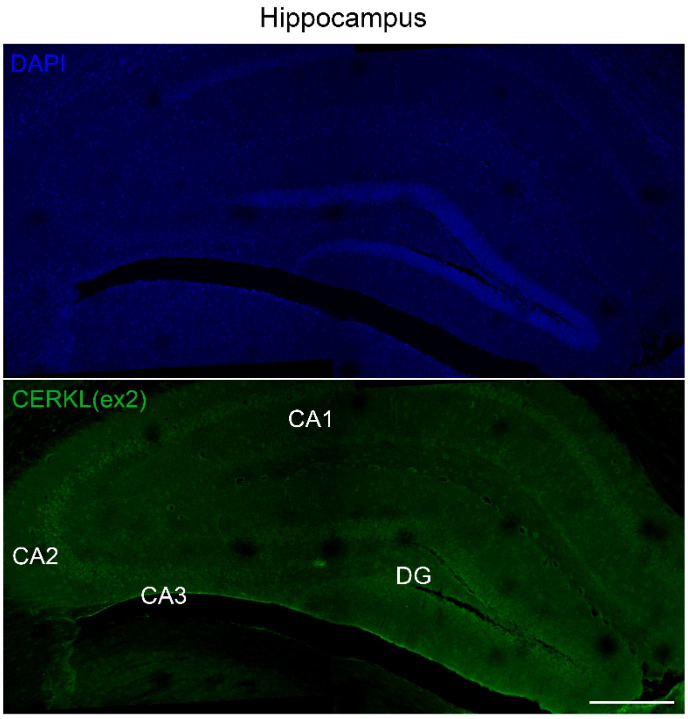

Figure S2. CERKL is expressed in adult mouse hippocampus. Adult mouse hippocampal cryosections immunostained with antibody CERKL(ex2) (green) and DAPI (blue), which stain the nuclei. CA1–CA3: Cornu Ammonis1–3; DG: dentate gyrus. Scale bar: 150 μm.

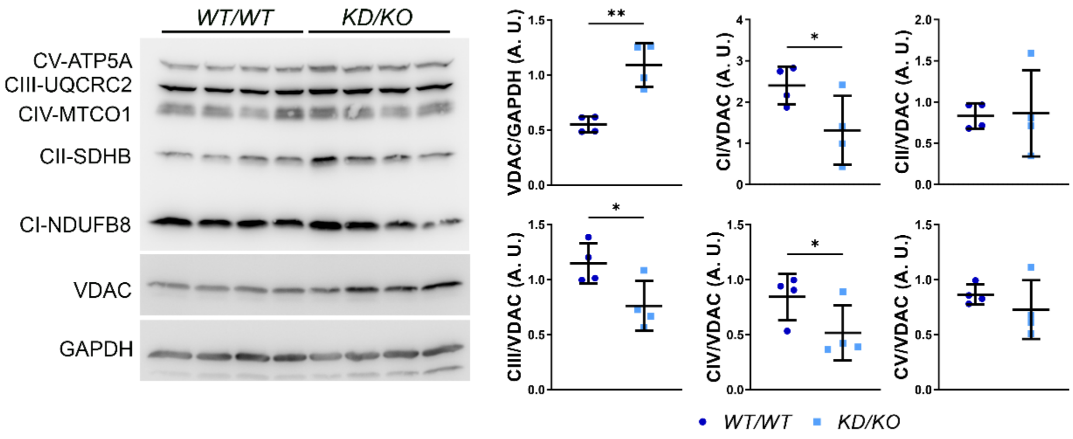

Figure S3. OXPHOS proteins are significantly altered in whole lysates from *KD/KO* hippocampi. Western blot analysis and quantification of OXPHOS proteins, VDAC and GAPDH in *WT/WT* and *KD/KO* hippocampi. VDAC is increased whereas CI-NDUFB8, CIII-UQCRC2 and CIV-MTCOI are decreased in *KD/KO* lysates, indicating shortage of OXPHOS proteins per mitochondria in *KD/KO* tissue. Statistical analysis by T-test. n=4 animals per genotype. *: p-value ≤0.5; **: p-value ≤0.01.
